# Tartary Buckwheat Extract Attenuated the Obesity-Induced Inflammation and Increased Muscle PGC-1a/SIRT1 Expression in High Fat Diet-Induced Obese Rats

**DOI:** 10.3390/nu11030654

**Published:** 2019-03-18

**Authors:** Seog-Young Kim, Mak-Soon Lee, Eugene Chang, Sunyoon Jung, Hyunmi Ko, Eunyoung Lee, Soojin Lee, Chong-Tai Kim, In-Hwan Kim, Yangha Kim

**Affiliations:** 1 Department of Nutritional Science and Food Management, Ewha Womans University, Seoul 03760, Korea; saraha9390@gmail.com (S.-Y.K.); troph@hanmail.net (M.-S.L.); eugenics77@hotmail.com (E.C.); cococosy@naver.com (S.J.); commi801@naver.com (H.K.); sheep1209@naver.com (E.L.); alicesujin@naver.com (S.L.); 2 R&D Center, EastHill Corporation, Gwonseon-gu, Suwon-si, Gyeonggi-do 16642, Korea; ctkim@ieasthill.com; 3 Department of Integrated Biomedical and Life Sciences, Korea University, Seoul 02841, Korea; k610in@korea.ac.kr

**Keywords:** tartary buckwheat extract, adipose tissue macrophage, obesity, inflammation, skeletal muscle, PGC-1α, SIRT1

## Abstract

Obesity is intimately related to a chronic inflammatory state, with augmentation of macrophage infiltration and pro-inflammatory cytokine secretion in white adipose tissue (WAT) and mitochondrial dysfunction in skeletal muscle. The specific aim of this study is to evaluate effects of tartary buckwheat extract (TB) on obesity-induced adipose tissue inflammation and muscle peroxisome proliferator-activated receptor-γ coactivator (PGC)-1α/sirtulin 1 (SIRT1) pathway in rats fed a high-fat diet. Sprague-Dawley rats were divided into four groups and fed either a normal diet (NOR), 45% high-fat diet (HF), HF + low dose of TB (TB-L; 5 g/kg diet), or HF + high dose of TB (TB-H; 10 g/kg diet) for 13 weeks. TB significantly reduced adipose tissue mass with decreased adipogenic gene expression of PPAR-γ and aP2. Serum nitric oxide levels and adipose tissue macrophage M1 polarization gene markers, such as iNOS, CD11c, and Arg1, and pro-inflammatory gene expression, including TNF-α, IL-6, and MCP-1, were remarkably downregulated in the TB-L and TB-H groups. Moreover, TB supplementation increased gene expression of PGC-1α and SIRT1, involved in muscle biogenesis and function. These results suggested that TB might attenuate obesity-induced inflammation and mitochondrial dysfunction by modulating adipose tissue inflammation and the muscle PGC-1α/SIRT1 pathway.

## 1. Introduction

Obesity refers to excessive fat accumulation in adipose tissue due to chronic positive energy balance [1]. Obesity status may play a role in the inflammatory response accompanied with metabolic diseases such as diabetes and cardiovascular disease. It has been shown that increase of fat accumulation contributes to the inflammatory mechanism, with changes in the secretion of pro-inflammatory cytokines [2,3,4].

Cytokines are secreted from adipose tissue macrophage (ATM), which classically comprise two different forms: pro-inflammatory macrophage M1 and anti-inflammatory macrophage M2. ATMs tend to change from M2 to M1 during the progression of adipose hypertrophy, and subsequently release pro-inflammatory cytokines such as tumor necrosis factor-α (TNF-α), interleukin 6 (IL-6), and monocyte chemoattractant protein 1 (MCP-1) [5,6]. Such transformation of adipose tissue-resident macrophages is referred as macrophage polarization.

In a nutrient-overloaded environment, dysregulation of mitochondrial biogenesis and dysfunctional mitochondria can result in incomplete mitochondrial fatty acid oxidation, increased reactive oxygen species (ROS) generation, and impaired glucose metabolism and insulin resistance [7,8,9]. These are supported by numerous clinical studies showing decreased activities of enzymes, biomarkers of muscle mitochondrial content, and mitochondrial number in obese human skeletal muscle [10,11,12]. Given the positive relationship between obesity and skeletal muscle mitochondrial dysfunction, it is necessary to prevent obesity-induced mitochondrial changes in skeletal muscle.

Buckwheat originated from the Yunnam province in southwest China and is widely consumed as flour or tea in many countries [13]. Buckwheat contains lots of bioactive components and is rich in flavonoids, including orientin, vitexun, quercetin, and rutin. Especially, tartary buckwheat (*Fagopyrum tataricum*) is known to have about 80-folds higher rutin than common buckwheat (*Fagopyrum esculentum*) [14]. From this respect, the bioactive functions of tartary buckwheat such as anti-oxidation, hypocholesterolemia, and anti-inflammation had been widely studied. Ethanol extract of tartary buckwheat sprout suppressed pro-inflammatory mediator production via inhibition of NF-κB p65 translocation in LPS-stimulated Raw 264.7 cells [15]. In 3T3-L1 cells, tartary buckwheat ethanol extract also attenuated inflammatory response with modulating nitric oxide (NO) production and gene expression of TNF-α, and IL-6 [16]. Therefore, this study investigated the effects of tartary buckwheat extract (TB) on adipogenesis regulation and inflammation and muscle mitochondrial biogenesis in a high-fat diet (HFD)-induced obese rat model.

## 2. Materials and Methods

### 2.1. Preparation of Tartary Buckwheat Extracts

The TB was kindly supplied by the SKBioland Co. (Ansan, Gyeonggi, Korea). The extraction was performed as previously described [16]. Briefly, the TB was extracted with 70% ethanol (1:15 (*w*/*v*)) at 80 °C for 3 h, and then filtered. The filtrate was vacuum-evaporated and the concentrated liquid was spray-dried into a powder form (Dongjin ENG Inc., Siheung, Korea).

### 2.2. Total Phenolic, Total Flavonoid, and Rutin Determination

Total phenolic content was determined using method from Folin-Denis [17]. In brief, 1 mL of TB was mixed with 0.5 mL of 2 N Folin-Ciocalteau reagent (Korean Food Standards Codex, 2011), and 2 mL of 10% sodium carbonate. The mixed solution was placed in a dark room for 90 min at room temperature, and then the absorbance was measured at 765 nm in a spectrophotometer (V-550, Jasco, Tokyo, Japan). Data was calculated with gallic acid (GAE) as standard, and expressed as mg of GAE equivalents per 1 g of dry weight.

Total flavonoids were determined using aluminum chloride method [18]. A total of 0.5 mL of tartary buckwheat extract was mixed with 0.5 mL of aluminum chloride (AlCl3), and incubated for 1 h at room temperature. The absorbance was measured at 420 nm in a spectrophotometer (V-550, Jasco, Tokyo, Japan). The concentration of total flavonoid was determined with quercetin standard curve, and expressed as mg of quercetin equivalents per 1 g of dry weight.

The rutin contents of the TB was analyzed with high-performance liquid chromatography (HPLC) and determined with rutin standard as previously described [16].

### 2.3. Animals and Experimental Design

All experimental procedures related to animals were approved by the Institutional Animal Care and Use Committee (IACUC) of Ewha Womans University (IACUC No. 16-022). Three-week-old male Sprague-Dawley rats, weighing 60–70 g each, were purchased from Doo Yeol Biotech (Seoul, Korea). Each rat was housed individually in a wire meshed cage under a controlled environment: 12-h light/dark cycle at a constant temperature (22 ± 2 °C) and humidity (55 ± 5%). After one week of acclimation, the rats were randomly divided into four groups (*n* = 8/group): normal diet (NOR), HFD (45% kcal from fat), HFD containing 0.5% TB extract (TB-L), and HFD containing 1.0% TB extract (TB-H). A commercial chow diet (Harlan 2018s; Harlan, Indianapolis, IN, USA) was served as NOR, which contained 18% crude protein, 6% fat, 44% carbohydrate, 15% fiber, and 5% ash. The diet composition of HFD, TB-L, and TB-H are described in Table 1. Body weight and food intake were measured twice a week during the 13-week experimental period. Rats were fasted for 16 h before sacrifice and anesthetized with zoletil:xylazine (4:1) at a dose of 0.1 mL/80 g body weight. Blood samples were collected by cardiac puncture and centrifuged at 1500× *g* for 20 min at 4 °C. Serum was obtained and stored separately at −40 °C until analysis. Epididymal white adipose tissue (WAT) and skeletal muscle (biceps femoris) were dissected, immediately frozen in liquid nitrogen, and stored at −70 °C until further analysis.

### 2.4. Serum Chemical Measurement

Serum levels of aspartate transaminase (AST), alanine transaminase (ALT), triglycerides (TG), total cholesterol (TC), and high-density lipoprotein cholesterol (HDL-C) were measured with enzymatic colorimetric methods using commercial kits (Asan Pharmaceutical Co., Ltd., Seoul, Korea). Low-density lipoprotein cholesterol (LDL-C) was calculated by the Friedewald formula: [LDL-C = TC − HDL-C − (TG/5)] [19].

### 2.5. Histological Analysis of Adipose Tissue

The epididymal WAT was fixed with a 10-fold volume of 10% formalin overnight. After fixing, the tissues were embedded in paraffin and sectioned and stained with hematoxylin and eosin (H&E) using a commercial staining kit (ScyTek Laboratories, Logan, UT, USA). Samples were observed under a microscope (Olympus, Tokyo, Japan) and captured at 200× magnification.

### 2.6. Immunohistochemistry of Adipose Tissue

Histological sections were processed as described above, deparaffinized, and rehydrated. The tissue sections were incubated with rabbit polyclonal anti-F4/80 (EGF-like module-containing, mucin-like, hormone receptor-like 1) (C2C3) antibody solution (GeneTex Inc., CA, USA), followed by solution from the Polink-2 Plus HRP anti-rat DAB detection kit (Golden Bridge International Inc. Irvine, CA, USA). The sections were stained with diaminobenzidine (Golden Bridge International, Inc.) and counterstained with Mayer hematoxylin (ScyTek).

### 2.7. Real-Time Quantitative Polymerase Chain Reaction (RT-qPCR)

Total RNA was isolated from epididymal WAT or skeletal muscle using Ribo Ex (Geneall Biotechnology Co., Ltd., Daejeon, Korea). The cDNA was synthesized from 4 μg total RNA using M-MLV reverse transcriptase (Bioneer Co., Daejeon, Korea). A fluorometric thermal cycler (Rotor GeneTM 2000; Corbett Research, Mortlake, N.S.W., Australia) and AccuPower 2X Greenstar qPCR Master mix (-ROX Dye) (Bioneer Co.) were utilized for Real Time qPCR analysis, and the data were relatively quantified using the ΔΔ*C*_t_ method [20]. Glyceraldehyde 3-phosphate dehydrogenase (GAPDH) was used as a reference gene for normalization and the results were expressed as a fold-difference compared to the HFD group. Primers used in the present study are described in supplementary Table S1.

### 2.8. Serum TNF-α Measurement

Serum TNF-α concentration was measured using Rat TNF-α ELISA MAX™ Deluxe (BioLegend, Inc., San Diego, CA, USA) according to manufacturer instructions. Serum TNF-α molecule density is expressed as a measure of enzyme-reacted color proportion. Absorbance of the samples was read using a microplate reader at 450 nm. The sensitivity of the kit was 2 pg/mL

### 2.9. Nitric Oxide (NO) Production Measurement

NO production was measured as serum nitrite using a commercial kit (Griess reagent kit for nitrate determination; Thermo Scientific, Pittsburgh, PA, USA). NO release into serum was reacted with the reagent provided by the kit for 30 min, and the absorbance was measured at 548 nm. The nitrite concentration was determined using sodium nitrite as a standard. Values were presented as fold-difference compared to values from the HFD group.

### 2.10. Glycerol-3-Phosphate Dehydrogenase (GPDH) Activity Analysis

GPDH activity was measured by using a GPDH assay kit (Takara, Japan) in WAT. Sample used for this analysis was obtained by homogenizing 0.1 g of WAT with 200 μL of enzyme extraction buffer. The assay procedure provided by the manufacturer was followed. Results were normalized to total protein concentration, which was determined with a bicinchoninic acid (BCA) protein assay kit (Thermo Scientific). Normalized GPDH activity was presented as the fold-difference compared to values from the HFD group.

### 2.11. Sirtulin (SIRT) and AMP-Activated Protein Kinase (AMPK) Activitivess

SIRT enzyme activity in extracted muscle nuclear proteins was measured using a colorimetric universal SIRT activity assay kit according to the manufacturer’s instructions (Abcam, Cambridge, MA, USA). In brief, nuclear fraction from skeletal muscle (biceps femoris) was extracted and purified using a nuclear extraction kit (Abcam). SIRT activity was measured by directly detecting SIRT-converted deacetylated products and normalized to their respective protein concentrations determined by BCA protein assay kit (Thermo Scientific).

AMPK activity was evaluated using a single-site, semi-quantitative immunoassay method—an AMPK kinase assay kit (MBL Life Science, Woburn, MA, USA) following the manufacturer’s instructions. As for the sample preparation, in brief, 0.1 g of epididymal WAT was homogenized in 400 μL RIPA buffer (ELPIS Biotech., Korea). After 10 min of incubation on ice, the solution was centrifuged (4 °C, 11,463× *g*, 20 min) and the aqueous phase was separated. Results were normalized to protein quantity determined with a BCA protein assay kit (Thermo Scientific). AMPK activity was normalized to protein concentration and expressed as the fold-difference compared to values from the HFD group.

### 2.12. Statistical Analysis

All data are expressed as mean ± standard error of the mean (SEM). Statistical analyses were performed using SPSS software (version 23; IBM Corporation, Armonk, NY, USA). Significant differences among different groups were analyzed by a one-way analysis of variance (ANOVA) followed by Tukey’s multiple comparison tests. *p* < 0.05 was considered significantly different.

## 3. Results

### 3.1. Effect of Tartary Buckwheat Extract on Body Fat Mass

First, we measured total polyphenol, total flavonoids, and rutin levels in TB using methods of Folin-Denis, aluminum chloride, and HPLC. As shown in Table 2, 1 g of dried TB contained 101.11 mg total phenolics, 95.05 mg total flavonoid, and 106.02 mg rutin.

### 3.2. Effect of Tartary Buckwheat Extract on Body Fat Mass

**Figure 1:**
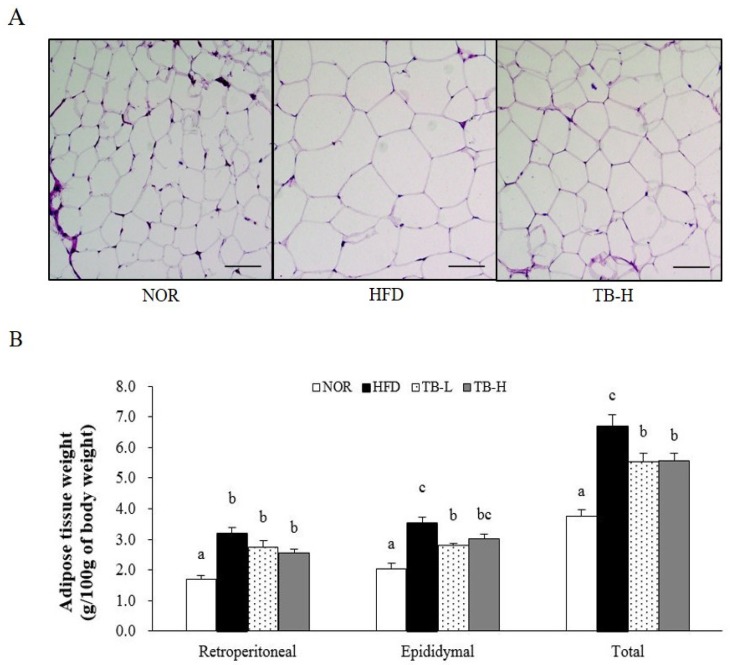
Effects of tartary buckwheat extract on white adipose tissue weight and adipocyte size. (**A**) Histology of adipose tissue. Representative images of hematoxylin and eosin (H&E) staining. Scale bars = 50 μm. (**B**) White adipose tissue weight. Data are expressed as mean ± SEM (*n* = 8). Values with different letters are significantly different at *p* < 0.05. NOR: normal diet; HFD: 45% high-fat diet; TB-L: HFD + 0.5% TB; TB-H: HFD + 1.0% TB.

After 13 weeks on the experimental diets, the final body weight of the rats consuming the HFD increased 1.15-fold compared to rats consuming the NOR diet. However, there was no statistical difference between the HFD group and the TB treatment groups (Table 3). Retroperitoneal, epididymal, and total WAT weights of rats in the HFD group were 86%, 71%, and 78% greater, respectively, than those in the NOR group (*p* < 0.05). TB treatment tended to reduce retroperitoneal WAT weight in a dose-dependent manner, but there was no statistical significance. Epididymal WAT weight in the TB-L group was significantly lower by 21% than in the HFD group (*p* < 0.05). In the TB-L and TB-H groups, total WAT weights were significantly lower by 18% and 16%, respectively, when compared to WAT mass in the HFD group (*p* < 0.05) (Figure 1B). Similar to total WAT mass, TB groups demonstrated smaller adipocyte size (Figure 1A).

### 3.3. Effect of Tartary Buckwheat Extract on Serum Lipid Profiles

The serum levels of TG, TC, HDL-C, and LDL-C are shown in Table 4. Serum TG levels in the TB-L and TB-H groups were significantly lower than the HFD group by 42% and 35%, respectively (*p* < 0.05). Serum TC levels of the TB-L and TB-H groups were also significantly decreased compared to the HFD group by 25% and 32%, respectively (*p* < 0.05). Moreover, rats in the HFD group exhibited lower serum HDL-C level by 22% compared to the NOR, and the TB-L group significantly improved HFD-decreased HDL-C concentration by 1.31-fold (*p* < 0.05) (Table 4).

### 3.4. Effect of Tartary Buckwheat Extract on Lipid Accumulation

**Figure 2:**
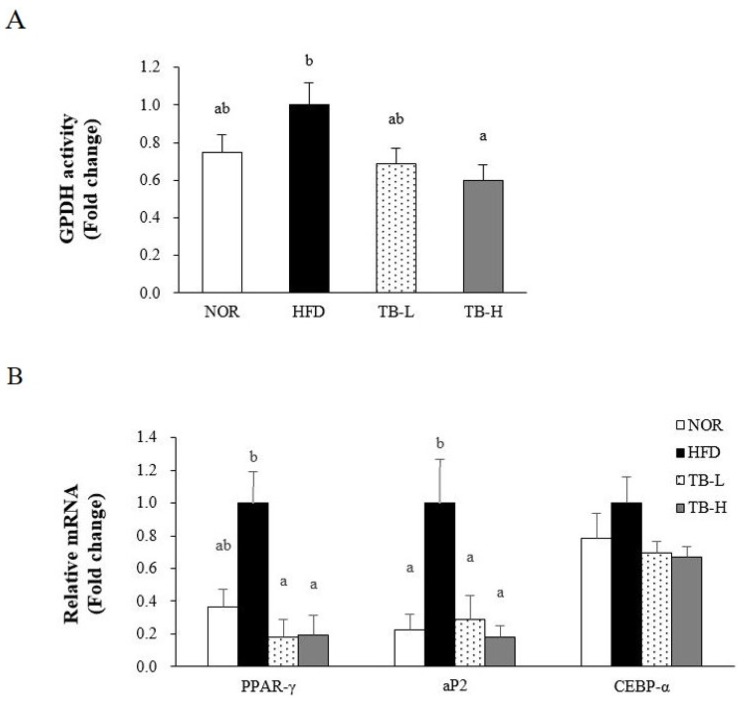
Effects of tartary buckwheat extract on adipogenesis. (**A**) Glycerol-3-phosphate dehydrogenase (GPDH) activity. (**B**) Gene expression level of PPAR-γ, ap2, and C/EBP-α. The mRNA level was normalized to GAPDH. Data are expressed as mean ± SEM (*n* = 8). Values with different letters are significantly different at *p* < 0.05. NOR: normal diet; HFD: 45% high-fat diet; TB-L: HFD + 0.5% TB; TB-H: HFD + 1.0% TB.

As TB treatment affected WAT weight and adipocyte size, enzyme activity and mRNA levels related to lipogenesis were investigated. TB attenuated GPDH activity in a dose-dependent manner, with a significant reduction evident at 1% TB (TB-H) compared to the HFD group (*p* < 0.05) (Figure 2A). Peroxisome proliferator activated receptor-γ (PPAR-γ), adipocyte fatty acid binding protein2 (aP2), and CCAAT/enhancer binding protein-alpha (C/EBP-α) were selected as adipogenesis-related genes (Figure 2B). The mRNA level of PPAR-γ was lower in the TB-L and TB-H groups than the HFD group by 82% and 80%, respectively (*p* < 0.05). TB-L and TB-H groups significantly decreased adipose aP2 mRNA expression, compared to the HFD group, by 71% and 82%, respectively (*p* < 0.05). Without statistical significance, both TB groups (TB-L and TB-H) downregulated C/EBP-α gene expression.

### 3.5. Effect of Tartary Buckwheat Extract on Macrophage Polarization in Adipose Tissue

**Figure 3:**
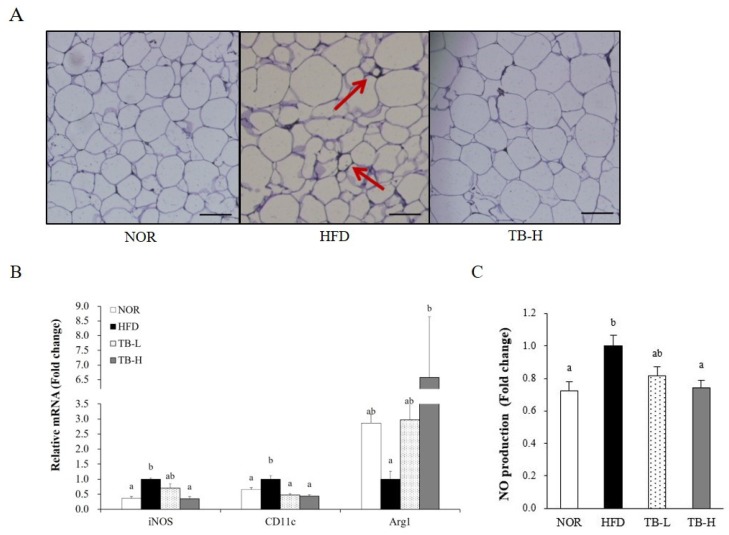
The effect of tartary buckwheat extract on adipose tissue macrophage infiltration and polarization. (**A**) F4/80 immunostaining of white adipose tissue, the arrows indicate crown-like structures. (**B**) mRNA level of M1 macrophage markers, inducible nitric oxide synthase (iNOS) and CD11c, and M2 macrophage marker, arginase 1 (Arg1). (**C**) NO production in rats fed experimental diets. Representative images of immunohistochemistry (IHC) images stained with F4/80. Scale bars = 50 μm. The mRNA level was normalized to glyceraldehyde 3-phosphate dehydrogenase (GAPDH). Data are expressed as mean ± SEM (*n* = 8). Values with different letters are significantly different at *p* < 0.05. NOR: normal diet; HFD: 45% high-fat diet; TB-L: HFD + 0.5% TB; TB-H: HFD + 1.0% TB.

We preformed further studies to confirm whether TB treatment could also alleviate adipose tissue macrophage infiltration and M1 polarization. F4/80 is a widely used macrophage marker and crown-like structures (CLSs) are developed when macrophages surround adipocytes. CLSs appeared in the HFD group, but were not observed in the NOR group or the TB-H group (Figure 3A), which suggests that TB treatment might ameliorate macrophage infiltration. Additionally, high treatment of TB (TB-H) significantly downregulated gene expression of M1 macrophage markers inducible nitric oxide synthase (iNOS) and CD11c by 65% and 56%, respectively, compared to the HFD group (*p* < 0.05). The mRNA level of the M2 macrophage maker arginase 1 (Arg1) was 6.58-fold higher in the TB-H group compared to the HFD group (*p* < 0.05) (Figure 3B). Similar to this, serum NO levels in the TB-H group were lower by 26% compared to the HFD group (*p* < 0.05) (Figure 3C).

### 3.6. Effect of Tartary Buckwheat Extract on Inflammatory Response

**Figure 4:**
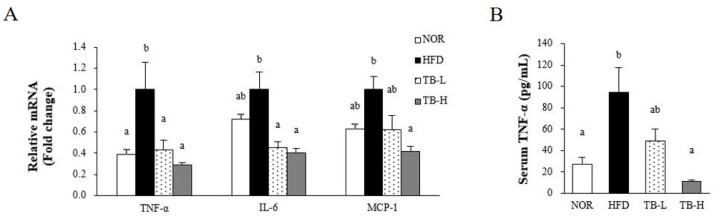
The effect of tartary buckwheat extract on pro-inflammatory cytokines. (**A**) mRNA level of pro-inflammatory factors TNF-α, IL-6, and MCP-1 in adipose tissue, and (**B**) serum TNF-α. The mRNA level was normalized to GAPDH. Data are expressed as mean ± SEM (*n* = 8). Values with different letters are significantly different at *p* < 0.05. NOR: normal diet; HFD: 45% high-fat diet; TB-L: HFD + 0.5% TB; TB-H: HFD + 1.0% TB.

We evaluated the effect of TB on adipocyte dysfunction by measuring serum TNF-α levels and gene expression of pro-inflammatory cytokines in WAT (Figure 4A,B). The mRNA levels of TNF-α in the TB-L and TB-H groups was lower by 57% and 71%, respectively, compared to the HFD group (*p* < 0.05). Both TB-L and TB-H significantly decreased IL-6 mRNA expression by 54% and 60%, respectively, compared to the HFD group (*p* < 0.05). Adipose MCP-1 mRNA level in the TB-H group was significantly decreased by 58% compared to the HFD group (*p* < 0.05). Similar to TNF-α expression, serum TNF-α levels were dose-dependently decreased by TB supplementation. The TB-H group showed 81% reduction in circulating TNF-α compared to the HFD group (*p* < 0.05).

### 3.7. Effect of Tartary Buckwheat Extract on AMPK Activity in Adipose Tissue

**Figure 5:**
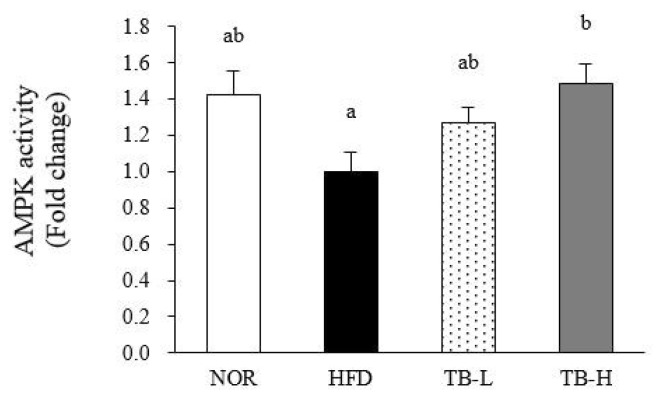
AMP-activated protein kinase (AMPK) activity in adipose tissue. AMPK activity was measured in white adipose tissue. Data are expressed as mean ± SEM (*n* = 8). Values with different letters are significantly different at *p* < 0.05. NOR: normal diet; HFD: 45% high-fat diet; TB-:, HFD + 0.5% TB; TB-H: HFD + 1.0% TB.

AMP-activated protein kinase (AMPK) is a key enzyme in the regulation of energy balance, concomitant adipogenesis, and the inflammatory response, and AMPK activity was measured herein in WAT. TB treatment significantly increased WAT AMPK activity in a dose-dependent manner with a 49% significant increment at 1% TB-supplemented group (TB-H) compared to the HFD group (*p* < 0.05) (Figure 5).

### 3.8. Effect of Tartary Buckwheat Extract on Mitochondrial Gene Expression and SIRT Activity in Skeletal Muscle

**Figure 6:**
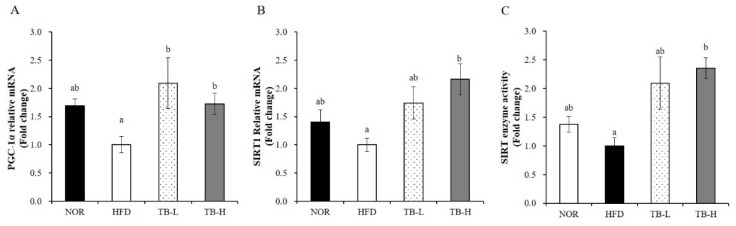
The effect of tartary buckwheat extract on muscle mitochondrial gene expression and sirtulin 1 (SIRT1) enzyme activity. The mRNA levels of PGC-1α (**A**) and SIRT1 (**B**) were analyzed by real-time PCR and normalized to GAPDH. SIRT enzyme activity was measured by a colorimetric SIRT activity assay kit (**C**). Data are expressed as mean ± SEM (*n* = 8). Values with different letters are significantly different at *p* < 0.05. NOR: normal diet; HFD: 45% high-fat diet; TB-L: HFD + 0.5% TB; TB-H: HFD + 1.0% TB.

Obesity is closely associated with mitochondrial loss and dysfunction. Next, we determined mRNA levels of mitochondrial genes such as peroxisome proliferator-activated receptor coactivator-1α (PGC-1α) and sirtulin 1 (SIRT1) in skeletal muscle. TB significantly upregulated muscle PGC-1α and SIRT1 gene expression about 2-fold compared to the HFD group (*p* < 0.05) (Figure 6A,B). In addition, TB treatment significantly increased muscle SIRT enzyme activity by up to 2.3-fold compared to HFD diet (Figure 6C). These data demonstrate that the favorable effect of TB on obesity may be in part due to mitochondrial change in skeletal muscle.

## 4. Discussion

Obesity refers to an abnormal adipose-hypertrophy state, which is often accompanied by a chronic inflammatory state [3,21]. The obesity-induced inflammatory response is intimately related with macrophage polarization [6]. Numerous in vivo and in vitro studies demonstrate that skeletal muscle of obese human subjects and rodents undergo mitochondrial changes and dysfunction, such as mitochondrial size and number and functional oxidation capacity [7,8,10,11,12,22]. Beneficial effects of tartary buckwheat on inflammatory response have been reported in macrophage cells and adipocyte [15,16]. The favorable effect of tartary buckwheat in inflammation, and its associated health outcomes, led us to investigate the effects of TB on high fat diet-induced adipose tissue inflammation via ATM infiltration and transformation and obesity-induced mitochondrial changes in skeletal muscle.

The currently used tartary buckwheat dosages was determined as described in previous studies with tartary buckwheat extract [23,24] and rutin, one of main substances in tartary buckwheat [24,25,26,27]. Used concentrations of 0.5% and 1.0% tartary buckwheat extract in the diet were well tolerated by rats in the present study, as demonstrated by no changes in liver weight and serum levels of AST and ALT. Serum lipid profile reflects a chronic obese state and, herein, serum lipid levels were measured to evaluate whether TB improved the serum lipid profile. The serum lipid profile appeared to be improved in the TB-treated groups, by lowering TG and TC and increasing HDL-C compared to the HFD group. Serum TG and TC results were similar to a previous study [23], which speculated that TB improves serum TG and TC via its anti-oxidation activity in hyperlipidemic rats, and might ultimately improve atheromatous plaque formation.

Without changing final body weight and food intake, in the present study, supplementation of tartary buckwheat extract to HFD (TB-L and TB-H) significantly decreased total WAT mass compared to HFD. H&E-stained WAT also revealed that the fat cell size was reduced in the TB treatment group. These observations suggested that TB treatment might alleviate high fat diet-induced adipocyte hypertrophy. The adipose tissue mass reduction itself has possible clinical significance because the adipose organ performs a key role in energy homeostasis and chronic inflammation by regulating lipid metabolism and cytokine secretion [21]. Further experiments were performed to investigate the efficacy of TB to restrain adipose tissue lipid accumulation and obesity-induced inflammation. PPAR-γ and C/EBP-α are adipogenic transcription factors that are key regulators of adipogenesis during adipocyte differentiation [28]. aP2 is a fatty acid carrier protein that plays an important role in fat accumulation and its gene expression level is usually used as an indicator of adipocyte differentiation [29]. In our study, the mRNA levels of PPAR-γ and aP2 were down-regulated by TB treatment. Increased expression of PPAR-γ also enhances GPDH activity, which consequentially stimulates TG synthesis in the cytosol [30]. From this aspect, we also investigated GDPH activity in WAT. Adipocyte GPDH is an enzyme that stimulates lipogenesis through the reduction of dihydroxyacetone phosphate into sn-glycerol-3 phosphate. Our experiment showed that GPDH activity in WAT was inhibited by the TB in a dose-dependent manner, and thus TB might suppress adipogenesis by regulating adipogenesis-related enzyme activity. Our study showed that TB has the possibility to attenuate adipocyte differentiation and TG deposition by downregulating adipogenic gene expression and adipogenesis-related enzyme activity. It has been reported that rutin, which is highly contained in buckwheat, blocks gene expression of PPAR-γ and aP2 in rats with HFD-induced obesity [27]. In 3T3-L1 adipocytes, rutin also has been demonstrated to inhibit adipogenic differentiation via inhibition of GPDH activity [23]. Taken together, it is likely that the rutin in the TB might be at least in part responsible for these results.

Adipocyte hypertrophy causes local adipose tissue hypoxia [31,32], which results in adipose tissue macrophage (ATM) infiltration [5] and polarization [6]. ATM infiltration is characterized by the development of CLSs, which occurs when macrophages surrounds adipocytes [33]. In the present study, we discovered CLSs presented in the HFD were diminished in TB-H group, suggesting TB treatment might play a role in adipocyte necrosis alleviation. Macrophage polarization is characterized in phenotypic switching of ATMs between pro-inflammatory M1 and anti-inflammatory M2 classes. M1 macrophage polarization in adipose tissue leads to an increase in inflammatory cytokine expression. iNOS, secreted from M1 macrophages, binds with calmodulin and produces NO molecules that work as inflammatory mediators [34]. Arginase 1 (Arg1), which is mainly secreted from M2 macrophages, inhibits iNOS activation [35,36]. CD11c is a surface molecule of M1 macrophages. In our study, mRNA expression of M1 macrophage markers iNOS and CD11c were downregulated and the M2 macrophage marker Arg1 was up-regulated with TB treatment. The modulation of iNOS and Arg1 expression might affect NO production. The NO production level measured in our study was consistent with the prior result, suggesting that TB treatment might inhibit ATM polarization to the M1 form. As rutin is reported to suppress adipose tissue M1 polarization in HFD-induced obese mice [37], we speculate that modulation of ATM transformation might be partially due to the activity of rutin. TNF-α and IL-6 are cytokines that are synthesized in ATM and increased with lipid accumulation; thus, these cytokines are indicators of obesity-associated inflammation [21]. MCP-1 is secreted from adipocytes and trigger macrophage clustering [21,38]. The mRNA levels of TNF-α, IL-6, MCP-1, and serum TNF-α were significantly decreased with TB treatment in our experiment. Likewise, tartary buckwheat sprout extract was reported to lower TNF-α, IL-6, and NO production levels in lipopolysaccharide-stimulated RAW264.7 cells [15]. AMPK is a key enzyme that regulates energy metabolism in response to the ATP/AMP ratio in the cytoplasm [39]. Chronic AICAR-induced AMPK activation promotes lipolysis in WAT [40] and PPAR-γ inhibition is attributed to this phenomenon. Recent studies have found that AMPK also plays an important role in the anti-inflammatory effect by increasing fatty acid oxidation in macrophages and inhibiting pro-inflammatory cytokine TNF-α [41]. AMPK regulation of pro-inflammatory cytokines TNF-α, IL-6, and MCP-1 is intimately related with M1 polarization [42]. AMPK reduced M1 polarization in a mutant model, which is explained with the up-regulation of pro-inflammatory cytokine expression and the iNOS/Arg1 ratio [43]. As our results showed that AMPK activity was dose-dependently increased with TB treatment, we speculate that the TB function of repressing adipose tissue inflammation might be related with AMPK activation.

Despite growing evidence showing the close relationship between obesity and muscle mitochondrial changes, the effect of tartary buckwheat extract on mitochondrial changes in skeletal muscle during the development of obesity has never been elucidated. Obesity-induced muscle mitochondria changes include reduced muscle mitochondria number and muscle oxidative capacity [8,11,12] and reduced expression in a cluster of nuclear genes responsible for oxidative metabolism (i.e., PGC-1α and genes activated by PGC-1α) [44,45]. As a master regulator of mitochondrial biogenesis, PGC-1α contributes to an increased mitochondrial ATP synthesis and oxidative metabolism, such as oxidative phosphorylation and fatty acid β-oxidation [46,47]. In post-translational modification, a nicotinamide adenine dinucleotide (NAD)-dependent deacetylase, SIRT1, is necessary for PGC-1α deacetylation. Given the importance of its acetylation status to PGC-1α activity, the interaction between SIRT1 and PGC-1α has been focused in metabolic regulation and mitochondrial biogenesis [48,49]. In the present study, TB supplementation significantly increased HFD-decreased gene expression of PGC-1α and SIRT1 and SIRT enzyme activity in skeletal muscle, which might partially underlie the favorable effect of TB on metabolic control in the PGC-1α/SIRT1 pathway.

Accordingly, we suggest that TB would ameliorate lipid metabolism and the inflammatory response partially, by modulating the activity of AMPK in adipose tissue and PGC-1α/SIRT1 signaling in skeletal muscle. AMPK activation might lead to a reduction in lipid storage by suppressing PPAR-γ mRNA expression and lowering GPDH activity and aP2 gene expression. Moreover, ATM infiltration and transformation toward the M1 form might be alleviated by AMPK, which ameliorates pro-inflammatory factors that are secreted from M1 macrophages, including TNF-α, IL-6, and MCP-1. In addition, the beneficial effects of TB might be implicated, at least in part, as TB-induced increases in muscle PGC-1α transcription and SIRT1 mRNA levels and enzyme activity.

## Figures and Tables

**Table 1:** The compositions of experimental diets (g/kg diet).

Component	HFD	TB-L	TB-H
Casein	238.79	238.79	238.79
Corn starch	185.11	180.11	175.11
Dextrose	157.60	157.60	157.60
Sucrose	59.70	59.70	59.70
Cellulose	59.70	59.70	59.70
Soybean oil	29.85	29.85	29.85
Lard	208.94	208.94	208.94
tert-Butylhydroquinone (TBHQ)	0.02	0.02	0.02
Mineral mix ^1^	41.79	41.79	41.79
Vitamin mix ^2^	11.94	11.94	11.94
L-cystine	3.58	3.58	3.58
Choline bitartrate	2.98	2.98	2.98
TB ^3^	-	5.00	10.00
Total	1000	1000	1000
Energy density (kcal/g)	4.8	4.8	4.8
Carb % (calories)	35	35	35
Fat % (calories)	45	45	45
Protein % (calories)	20	20	20

^1^ AIN-93G Mineral Mix; ^2^ AIN 93G Vitamin Mix; ^3^ TB, abbreviation of tartary buckwheat extract. HFD: 45% high-fat diet; TB-L: HFD + 0.5% TB; TB-H: HFD + 1.0% TB.

**Table 2:** Bioactive compounds found in tartary buckwheat extracts.

Contents	Tartary Buckwheat Extract
Total phenolics (mg GAE/g dried sample)	101.11 ± 5.5
Total flavonoid (mg QE/g dried sample)	95.05 ± 3.6
Rutin (mg/g dried sample)	106.02 ± 1.3

Data are expressed as mean ± SEM. Total phenolic contents were expressed as mg of gallic acid (GAE) equivalents per 1 g of dry weight. The concentrations of total phenolics, total flavonoid, and rutin were expressed as mg of gallic acid (GAE), quercetin (QE), and rutin equivalents per 1 g of dry weight.

**Table 3:** Effect of tartary buckwheat extract on body weight and food intake.

Variables	NOR	HFD	TB-L	TB-H
Initial body weight (g)	67.24 ± 2.16 ^a^	66.16 ± 2.23 ^a^	66.42 ± 2.31 ^a^	66.90 ± 2.17 ^a^
Final body weight (g)	482.46 ± 16.86 ^a^	554.49 ± 12.52 ^b^	556.47± 19.08 ^b^	555.42 ± 13.26 ^b^
Body weight gain (g/13 weeks)	415.22 ± 17.49 ^a^	488.33 ± 11.32 ^b^	490.05 ± 19.87 ^b^	488.52 ± 12.25 ^b^
Food intake (g/day)	25.59 ± 1.62 ^a^	21.02 ± 0.54 ^a^	20.28 ± 0.53 ^a^	20.17 ± 0.50 ^a^
Energy intake (kcal/day)	79.33 ± 5.01 ^b^	100.49 ± 2.58 ^a^	96.95 ± 2.53 ^a^	96.41 ± 2.40 ^a^
Energy efficiency ratio (EER) ^1^	0.058 ± 0.004 ^a^	0.053 ± 0.000 ^a^	0.055 ± 0.001 ^a^	0.056 ± 0.000 ^a^

^1^ Energy efficiency = bodyweight gain (g/day)/Energy intake (g/day); data are expressed as mean ± SEM (*n* = 8). Values with different letters are significantly different at *p* < 0.05. NOR: normal diet; HFD: 45% high-fat diet; TB-L: HFD + 0.5% TB; TB-H: HFD + 1.0% TB.

**Table 4:** Effect of tartary buckwheat extract on serum lipid profiles.

Variables	NOR	HFD	TB-L	TB-H
Serum lipids (mmol/L)				
Triglyceride	0.58 ± 0.04 ^ab^	0.73 ± 0.07 ^b^	0.43 ± 0.03 ^a^	0.48 ± 0.07 ^a^
Total cholesterol	2.79 ± 0.17 ^bc^	2.98 ± 0.16 ^c^	2.24± 0.13 ^ab^	2.03 ± 0.13 ^a^
HDL cholesterol	1.59 ± 0.08 ^b^	1.23 ± 0.07 ^a^	1.63 ± 0.07 ^b^	1.46 ± 0.02 ^ab^
LDL cholesterol	1.06 ± 0.12 ^b^	1.50 ± 0.11 ^b^	0.52 ± 0.10 ^a^	0.56 ± 0.12 ^a^
Liver toxicity (IU/L)				
AST	38.96 ± 2.17	40.10 ± 3.61	37.91 ± 3.38	37.99 ± 2.64
ALT	8.23 ± 0.52	8.06 ± 0.64	6.86 ± 0.80	7.11 ± 0.32

Data are expressed as mean ± SEM (*n* = 8). Values with different letters are significantly different at *p* < 0.05. NOR: normal diet; HFD: 45% high-fat diet; TB-L: HFD + 0.5% TB; TB-H: HFD + 1.0% TB; HDL: high-density lipoprotein cholesterol; LDL: low-density lipoprotein cholesterol; AST: aspartate aminotransferase; ALT: alanine transaminase.

## Supplementary Materials

The following are available online at https://www.mdpi.com/2072-6643/11/3/654/s1, Table S1: Primers used for quantitative real time PCR.
