# Supplementary material for: Tartary Buckwheat Extract Attenuated the Obesity-Induced Inflammation and Increased Muscle PGC-1a/SIRT1 Expression in High Fat Diet-Induced Obese Rats
Source: Nutrients. 2019 Mar 18;11(3):654. doi: 10.3390/nu11030654 (PMC6471111; doi:10.3390/nu11030654)
Supplement: Supplementary file 1 [file nutrients-11-00654-s001.pdf]

nutrients

MDPI

Table S1. Primers used for quantitative real time PCR

Name	GeneBank No.	Primer sequence (5′-3′)
aP2	NM_053365	F: TCACCCCAGATGACAGGAAA<br>R: CATGACACATTCCACCACCA
Arg1	NM_017134.3	F: ACATCGGCTTGCGAGATGTG<br>R: GCCAATTCCCAGCTTGTCCA
C/EBP-α	NM_012524	F: GCCAAGAAGTCGGTGGATAA<br>R: CGGTCATTGTCACTGGTCAA
CD11c	XM_006230382.3	F: CAGAACCCGTCCACCCAATG<br>R: GATGTCACAGCGGAAGTGCA
GAPDH	NM_017008	F: ACCACAGTCCATGCCATCAC<br>R: TCCACCACCCTGTTGCTGTA
IL-6	NM_012589	F: ATAGTCCTTCCTACCCCAAC<br>R: TGCCGAGTAGACCTCATAGT
iNOS	NM_012611.3	F: TCCTGCCACCTTGGAGTTCA<br>R: TGGTCACCTCCAGCACAAGA
MCP-1	NM_031530	F: ACTCACCTGCTGCTACTCAT<br>R: CTACAGCTTCTTTGGGACAC
PGC-1α	NM_031347	F: GCACCAGAAAACAGCTCCAA<br>R: TTACTGAAGTTGCCATCCCG
PPAR-γ	NM_001145366	F: TGTGGGGATAAAGCATCAGG<br>R: CAAGGCACTTCTGAAACCGA
SIRT1	XM_008772947.2	F: AGGGAACCTCTGCCTCATCT<br>R: GAGGTGTTGGTGGCAACTCT
TNF-α	NM_012675	F: CCCCTTTATCGTCTACTCCT<br>R: ACTACTTCAGCGTCTCGTGT

aP2, adipocyte protein 2; Arg1, arginase 1; C/EBP-α, CCAAT/enhancer binding protein-α; GAPDH, glyceraldehyde-3-phosphate dehydrogenase; IL-6, interleukin-6; iNOS, inducible nitric oxide synthase; MCP-1, monocyte chemoattractant protein-1; PGC-1α, peroxisome proliferative activated receptor gamma coactivator-1α; PPAR-γ, peroxisome proliferator-activated receptor-γ; SIRT1, sirtuin 1; TNF-α, tumor necrosis factor-α.
